# Optimal Scheduling of Microgrids Based on a Two-Population Cooperative Search Mechanism

**DOI:** 10.3390/biomimetics10100665

**Published:** 2025-10-01

**Authors:** Liming Wei, Heng Zhong

**Affiliations:** School of Electrical and Computer Engineering, Jilin Jianzhu University, Changchun 130118, China; weiliming@jlju.edu.cn

**Keywords:** IMOHHOGWO, multi-objective optimization, Harris Hawk Optimizer, Grey Wolf Optimizer, carbon emission cost, Simulated Annealing (SA) strategy, optimized scheduling of microgrids

## Abstract

Aiming at the problems of high-dimensional nonlinear constraints, multi-objective conflicts, and low solution efficiency in microgrid optimal scheduling, this paper proposes a multi-objective Harris Hawk–Grey Wolf hybrid intelligent algorithm (IMOHHOGWO). The problem of balancing the global exploration and local exploitation of the algorithm is solved by introducing an adaptive energy factor and a nonlinear convergence factor; in terms of the algorithm’s exploration scope, the stochastic raid strategy of Harris Hawk optimization (HHO) is used to generate diversified solutions to expand the search scope, and constraints such as the energy storage SOC and DG outflow are finely tuned through the α/β/δ wolf bootstrapping of the Grey Wolf Optimizer (GWO). It is combined with a simulated annealing perturbation strategy to enhance the adaptability of complex constraints and local search accuracy, at the same time considering various constraints such as power generation, energy storage, power sales, and power purchase. We establish the microgrid multi-objective operation cost and carbon emission cost objective function, and through the simulation examples, we verify and determine that the IMOHHOGWO hybrid intelligent algorithm is better than the other three algorithms in terms of both convergence speed and convergence accuracy. According to the results of the multi-objective test function analysis, its performance is superior to the other four algorithms. The IMOHHOGWO hybrid intelligent algorithm reduces the grid operation cost and carbon emissions in the microgrid optimal scheduling model and is more suitable for the microgrid multi-objective model, which provides a feasible reference for future integrated microgrid optimal scheduling.

## 1. Introduction

As a critical infrastructure for integrating distributed energy resources, energy storage systems, and electrical loads, optimal scheduling of microgrids represents a fundamental challenge in achieving efficient energy utilization and low-carbon operation. The increasing penetration of renewable energy sources and the evolving complexity of electricity market mechanisms have transformed microgrid scheduling into a multi-timescale, multi-objective optimization problem characterized by high-dimensional constraints [1]. Traditional single-objective optimization approaches prove inadequate in addressing the simultaneous optimization of conflicting objectives, including economic efficiency, environmental sustainability, and system reliability [2]; multi-objective optimization algorithms have emerged as a prominent research focus due to their superior capabilities in Pareto frontier exploration [3].

The integration of renewable energy sources presents several key challenges for multi-objective optimal scheduling in microgrids: (1) the complexity of power balance constraints arising from the inherent uncertainty of wind and solar generation [4]; (2) the need for simultaneous optimization of conflicting objectives encompassing economic, environmental, and reliability considerations [5,6]; and (3) the trade-off between convergence efficiency and solution set distribution in high-dimensional solution spaces [7,8]. Conventional optimization techniques, such as linear programming and dynamic programming, demonstrate limited effectiveness in addressing these nonlinear multi-objective problems, thereby necessitating the development of advanced metaheuristic algorithms [9].

**Figure 1:**
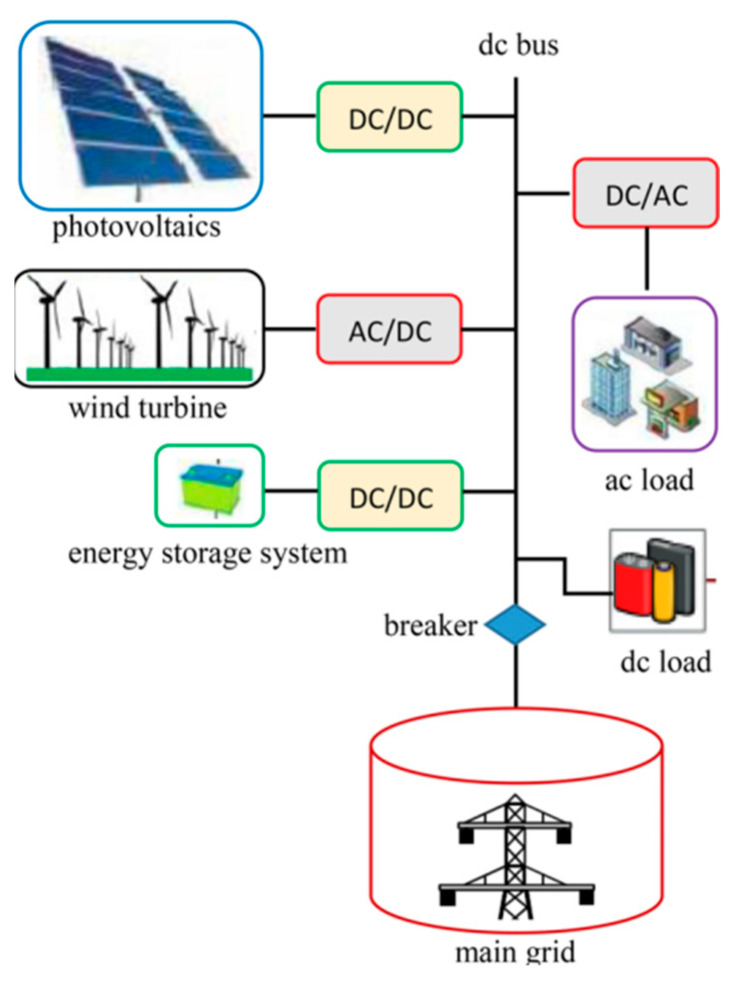
Microgrid architecture.

Microgrids constitute an essential component of smart grid infrastructure, as illustrated in Figure 1. These systems serve as effective platforms for integrating distributed generators (DGs), loads, energy storage devices, and control units, thereby maximizing the economic, environmental, and operational benefits of distributed generation while ensuring high-quality power delivery and meeting stringent reliability requirements. Furthermore, microgrids exhibit exceptional operational flexibility, capable of functioning in both grid-connected and islanded modes [10]. This dual-mode capability enables a continuous power supply to critical loads during grid disturbances. The operation of microgrid systems must, therefore, balance three fundamental objectives: economic efficiency, system reliability, and energy conservation.

## 1.1. Related Work

Metaheuristic algorithms, like HHO, have emerged as powerful tools for tackling such complex problems. HHO is a metaheuristic algorithm inspired by the hunting behavior of Harris hawks. It has gained popularity due to its flexibility, robustness, and scalability. Several studies have explored its application in various optimization problems, including job-shop scheduling, feature selection, and power control in microgrids.

Despite its advantages, the standard HHO can suffer from premature convergence and stagnation in local optima, especially when dealing with complex, nonlinear problems. This limitation has motivated the development of improved HHO variants. For instance, Liu [11] proposed an IHHO where the position of Harris hawks during global search is not random but is guided by the position of the best hawks in the population, effectively enhancing candidate solution quality. Kumar et al. [12] employed CHHO for EV charging scheduling, demonstrating improved performance in reducing charging time and optimizing charging station allocation. Shaker et al. [13] proposed a multi-objective hunger game search optimizer (MOHGS) for hybrid microgrid scheduling, considering multiple objectives such as minimizing operating costs and emissions and ensuring an uninterruptible power supply. Al-Shamma’a et al. [14] applied HHO to determine the optimal sizing of a hybrid renewable microgrid, aiming to minimize the annualized system cost. The performance of HHO was compared with other metaheuristic techniques, demonstrating its effectiveness. Choo et al. [15] designed an ensemble-based Harris Hawk Optimizer (EN-HHO) to address the multi-objective flexible job shop problem (MOFJSP). Rajagopalan et al. [16] presented a new optimization algorithm, i.e., the Oppositional Gradient-Based Grey Wolf Optimizer (OGGWO), to elucidate the optimal operation in microgrids that are loaded with sustainable, as well as unsustainable, energy sources. Tang et al. [17] developed an ordered charging and discharging optimization scheduling strategy for energy storage charging piles considering time-of-use electricity prices based on the Multi-Strategy Hybrid Improved Harris Hawk Algorithm (MHIHHO).

Among these techniques, the GWO has been notably employed due to its robustness and efficiency in handling multi-objective problems. Du et al. [18] integrated HHO with the GWO, fuzzy C-means (FCM) clustering, the Particle Swarm Optimizer (PSO), and the Bat Optimizer (BO) to create an Improved Grey Wolf Optimizer (IGWO) for optimal scheduling of integrated energy systems. This approach aims to leverage the strengths of different algorithms to enhance the overall performance. Makhadmeh et al. [19] hybridized the GWO with a Min-Conflict Algorithm (MCA) to enhance power scheduling in smart homes, formulating the problem as a multi-objective optimization task. This hybrid approach aimed to improve solution quality and convergence speed, emphasizing the GWO’s potential in residential energy management. Rajagopalan et al. [20] introduced an Oppositional Gradient-Based Grey Wolf Optimizer (OGGWO). Their study focused on optimizing microgrid operations with diverse energy sources, validating the algorithm across multiple scenarios to demonstrate its efficacy and robustness in achieving optimal energy utilization.

In the broader context of multi-objective optimization, other algorithms such as Non-Dominated Sorting Dung Beetle Optimization [21] and the Hunger Game Search Optimizer [22] have been explored for microgrid scheduling, but GWO remains prominent due to its balance of exploration and exploitation capabilities, as evidenced by its successful applications across various energy and manufacturing scheduling problems.

From the above summary, it can be seen that the HHO algorithm and the GWO algorithm each have their own advantages and disadvantages. Drawing inspiration from both algorithms, we innovatively combined them and further integrated them with the simulated annealing perturbation strategy to generate a hybrid algorithm, IMOHHOGWO. This algorithm inherits the raid strategy of the HHO algorithm and the social hierarchy guidance of the GWO algorithm, combining their strengths and compensating for their weaknesses. In subsequent test function experiments, the significant advantages of IMOHHOGWO are evident.

## 1.2. Contributions

### 1.2.1. Algorithmic Innovation and Hybrid Framework Development

- Novel Hybrid Multi-Objective Algorithm: This paper introduces IMOHHOGWO, which innovatively combines the dynamic raid strategy of HHO with the social hierarchy mechanism of the GWO. This hybrid approach leverages the complementary strengths of both algorithms—HHO’s superior global exploration capabilities through stochastic raid strategies and the GWO’s efficient local exploitation through α/β/δ wolf leadership guidance.
- Advanced Adaptive Parameter Mechanisms: The algorithm incorporates several cutting-edge improvements, including (1) a nonlinear convergence factor that replaces traditional linear approaches with exponential forms for enhanced convergence speed; (2) a diversity perception energy factor that dynamically adjusts based on population standard deviation to balance exploration and exploitation; and (3) adaptive energy factor control that responds to solution set distribution density.
- Enhanced Global–Local Search Balance: The fusion strategy successfully addresses the fundamental challenge of balancing global exploration and local exploitation in multi-objective optimization through dynamic strategy switching controlled by escape energy factors and leadership hierarchy guidance.
- Lévy Flight Integration: Incorporates Lévy flight mechanics during exploitation phases to enable strategic long-distance jumps, significantly improving the algorithm’s ability to escape local optima and explore promising solution regions in high-dimensional search spaces.
- Simulated Annealing Integration: A temperature decay perturbation mechanism is strategically integrated during the exploitation phase with 0.3 probability, significantly enhancing the algorithm’s ability to escape local optima and improve solution quality in complex constraint environments.

### 1.2.2. Mathematical Modeling and Problem Formulation Excellence

- Comprehensive Microgrid Mathematical Framework: This establishes detailed mathematical models for all distributed generation units, including wind turbines, photovoltaic systems, micro-gas turbines, diesel generators, and energy storage batteries, incorporating realistic operational constraints, efficiency factors, and environmental parameters that accurately reflect real-world microgrid operations.
- Dual-Objective Optimization Model: This develops a sophisticated multi-objective mathematical model that simultaneously optimizes economic operating costs and carbon emission costs, addressing the critical trade-off between economic efficiency and environmental sustainability in microgrid operations. The model incorporates fuel costs, depreciation costs, maintenance costs, and comprehensive pollutant treatment costs (CO_2_, SO_2_, and NO_2_).
- Advanced Constraint Handling: Implements comprehensive constraint management, including power balance constraints, distributed generation output limits, energy storage SOC constraints, and grid interaction limits, ensuring the practical feasibility of optimization solutions.

### 1.2.3. Superior Performance Validation and Comparative Analysis

- Exceptional Multi-Objective Test Performance: Comprehensive evaluation using standard ZDT1, ZDT2, and ZDT3 test functions demonstrates IMOHHOGWO’s superiority across all metrics. IMOHHOGWO consistently obtained the best GD values and achieved the highest hypervolume (HV) values among all compared algorithms while maintaining competitive solution distribution uniformity.
- Robust Algorithm Comparison: Systematic comparison with four state-of-the-art algorithms (MODBO, MOPSO, MOHHO, and NSGA-II) across multiple performance metrics validates the consistent superiority of the proposed approach in terms of convergence accuracy, solution quality, and computational efficiency.

### 1.2.4. Application Impact and Innovation

- Practical Microgrid Implementation: This successfully validates the algorithm using real microgrid data with 24 h load profiles, actual equipment parameters, real-time electricity pricing, and authentic operational constraints, demonstrating annual operating cost savings of RMB 37,669.6 and carbon emission reduction of 0.449 tons.
- Multi-Scenario Optimization Capability: This provides comprehensive analysis across three optimization scenarios (economic dispatch, environmental dispatch, and multi-objective scheduling), offering decision-makers flexible tools to balance economic and environmental objectives based on specific operational requirements and policy constraints.
- Advanced Energy Management Strategy: The algorithm enables sophisticated energy management through optimal scheduling of diverse distributed generation sources, intelligent energy storage utilization, and strategic grid interaction, significantly improving overall microgrid efficiency and sustainability.

This comprehensive contribution framework systematically addresses the gap between traditional single-objective optimization approaches and the complex, multi-faceted requirements of modern sustainable microgrid operations, providing a robust foundation for future intelligent energy management system development.

## 2. Microgrid Power Systems with Renewable Energy

### 2.1. Modelling of Distributed Power for Microgrids

#### 2.1.1. Wind Turbine Model (WT)

The output power of the wind power generation unit is directly related to the value of the wind speed, and the fluctuation of its output characteristics is more obvious [23]. Its mathematical model is shown in Equation (1):

(1) $$P_{wt}=\left\{\begin{array}{l} 0,\;v<v_{ci}orv\geq v_{co}\\ \frac{v^{3}-v_{ci}^{3}}{v_{r}^{3}-v_{ci}^{3}},v_{ci}\leq v\leq v_{r}\\ P_{r},v_{r}\leq v\leq v_{co} \end{array}\right.$$

In Equation (1), v_ci_, v_r_, and v_co_, respectively, are the generator’s cut-in wind speed, rated wind speed, and cut-out wind speed (in this paper, respectively, 3 m/s, 14 m/s, and 25 m/s); P_wt_ is the actual power of the wind turbine; and Pris is the rated power of the wind turbine.

#### 2.1.2. Photovoltaic Power Generation (PV)

According to the output characteristics of solar energy [24], we establish the following mathematical model of the power output of the PV power system, as shown in Equation (2):

(2) $$P_{pv}=R_{pv}q_{pv}\frac{I_{t}}{I_{STC}}[1+k(T_{C}-T_{stc})]$$

In Equation (2), P_pv_ is the PV cell output power, R_pv_ is the PV cell PV output power under standard test conditions, q_pv_ is the PV derating coefficient of 0.8, I_t_ is the actual solar radiation intensity, I_STC_ is the solar radiation intensity under standard test conditions, k is the temperature coefficient of the PV panels, T_c_ is the temperature of the PV panels at the current time step, and T_stc_ is the temperature of the PV panels under standard test.

#### 2.1.3. Diesel Electric Generator (DE)

A diesel generator is a commonly used fuel generator [25], and its fuel cost is its consumption characteristic function, as shown in Equation (3):

(3) $$\left\{\begin{array}{l} C_{DE}(t)=\alpha P_{DE}^{2}(t)+\beta P_{DE}(t)+\gamma \\ \alpha =0.00085,\beta =0.12,\gamma =6 \end{array}\right.$$

In Equation (3), C_DE_ (t) is the fuel cost of the diesel generator at time t; P_DE (t)_ is the output power of the diesel generator at time t; and α, β, and γ are the fuel cost coefficients of the diesel generator.

#### 2.1.4. Micro-Gas Turbine (MT)

The micro-gas turbine generates electricity by combusting the material [26], which has the advantages of fast response time, self-adjustable output power, etc., and the mathematical model is shown in (4) and (5):

(4) $$F_{MT}=C\frac{1}{LHV}\frac{P_{MT}}{\eta_{MT}}$$

(5) $$\eta_{MT}(t)=0.0753{\left[\frac{P_{MT}(t)}{65}\right]}^{3}-0.3095{\left[\frac{P_{MT}(t)}{65}\right]}^{2}+0.4174\frac{P_{MT}(t)}{65}+0.1068$$

F_MT_ is the fuel cost of the MT, and C is the price of natural gas; this paper uses RMB 2/m^3^. LHV is the low calorific value of natural gas; this paper takes 9.7 KWh/m^3^. P_MT_ (t) is the active output power of the micro-gas turbine. η_MT_ (t) is the micro-gas turbine operating efficiency, its value, and the cubic function of the relationship.

#### 2.1.5. Energy Storage Battery (BESS)

As a kind of energy storage equipment to ensure the safety and stability of microgrid system [27], a battery can track the change in wind energy and solar energy output for charging and discharging—playing a role in the grid to buffer the uncertainty of wind energy and solar energy output—and improves the reliability and continuity of the power supply of the grid. Its charging and discharging point mathematical model is shown below:

(6) $$SOC(t)=\left\{\begin{array}{l} SOC(t-1)+\frac{1}{\eta^{-}}P_{bess}(t),P_{bess}(t)\leq 0\\ SOC(t-1)+\eta^{+}P_{bess}(t),P_{bess}(t)>0 \end{array}\right.$$

In Equation (6), SOC (t) is the remaining capacity of the battery at time t; P_bess_ (t) is the charging and discharging power of the battery at time t, positive for charging, negative for discharging; η^+^ is the charging efficiency; and η^−^ is the discharging efficiency.

Establishing a mathematical model for microgrid power generation units can accurately predict and optimize distributed power output and reduce operating costs. For example, the photovoltaic power generation model takes into account the influence of light and temperature, and the wind power generation model takes into account the influence of wind speed. By establishing a mathematical model, it is possible to optimize the distributed power output, predict the load changes, optimize the energy scheduling, improve the economic efficiency and operational efficiency, control the output power in real time, maintain the stability of the voltage and frequency, and realize precise control.

### 2.2. Multi-Objective Mathematical Model for Optimal Microgrid Scheduling

The microgrid is in grid-connected mode, and under its system constraints [28], the economic reliability and environmental protection of the microgrid are comprehensively considered to establish a multi-objective optimal scheduling model for the microgrid system with the lowest operating costs and the lowest carbon emissions.

#### 2.2.1. Operating Costs

The generation costs of the microgrid mainly comprise the operation costs and the compensation costs of the interruptible load [29], so the generation cost minimizes the operation costs of the microgrid under the system equation constraints and inequality constraints.

(7) $$\min F=\sum_{t=1}^{T}\sum_{i}^{N}f_{i,t}(P_{i,t})+M_{t}$$

In Equation (7), T is the number of time slots in the scheduling cycle of the microgrid; N is the number of distributed power supply types; f_i,t_ is the generation cost of distributed power supply i at time t; M_t_ is the interruptible cost of the microgrid at time t; and P_i,t_ is the generation power of the ith microgrid at time t.

##### Power Generation Cost

The microgrid operating costs mainly comprise the unit’s fuel cost [30], depreciation costs, and maintenance costs; because PV and WT are clean energy, in the operation process, they will not consume fossil fuels, so we do not consider the PV and WT fuel costs, namely,

(8) $${\mathrm{CO}}_{i,t}={\mathrm{FC}}_{i,t}\left(P_{i,t}\right)+de_{i,t}\left(P_{i,t}\right)+{\mathrm{MC}}_{i,t}\left(P_{i,t}\right)$$

In Equation (8), FC _i,t_ is the fuel cost of distributed power supply i at time t; de_i,t_ is the depreciation cost of micropower supply i discounted to the unit time; and MC_i,t_ is the maintenance cost of micropower supply i at time t.

##### Depreciation Expenses


(9) $${IV}_{i,t}=\frac{C_{IC,i}}{8760\times P_{n,i}\times f_{m,i}}\times \frac{\alpha (1+\alpha {)}^{N}}{(1+\alpha {)}^{N}-1}$$

In Equation (9), C_IC,I_ is the installation cost of the ith distributed power supply; P_n,i_ is the power rating of the ith micropower supply; f_c,i_ is the capacity factor of the ith micropower supply; α is the interest rate or depreciation rate; and N is the lifetime of the micropower supply.

##### Maintenance Cost


(10) $${\mathrm{OM}}_{i,t}=K_{m,i}\times P_{i,t}$$

In Equation (10), K_m,i_ is the unit O&M cost for distributed power i.

#### 2.2.2. Cost of Carbon Emissions

The carbon emission cost mainly considers the emission treatment cost of the unit [31], as well as the PV and WT. Since PV and WT are clean energy sources and do not produce pollutant gases during operation, the carbon emission cost of PV and WT is not considered. With the objective function of minimizing carbon emission cost, the expression is

(11) $$\min C=\sum_{i=1}^{T}\sum_{j=1}^{K}\alpha_{j}\sum_{i=1}^{N}\beta_{ij}P_{i,t}$$

In Equation (11), C is the cost of carbon emission; K is the type of pollutant emission (CO_2_, SO_2_, and NO_2_); α_j_ is the unit cost of treating the jth pollutant, RMB/kg; and β_ij_ is the emission coefficient of the jth pollutant emitted at the time of outputting electricity under the different methods of electricity production, g/(kW-h).

#### 2.2.3. Constraints

Micropower Output Constraints

(12) $$P_{i,\min}\leq P_{i}\leq P_{i,\max}$$

In Equation (12), P_i,min_ and P_i,max_, respectively, are the lower and upper limits of the micropower output.

Microgrid power balance constraints:

(13) $$\sum_{i=1}^{N}P_{i}+P_{IL}=P_{L}-P_{RS}$$

In Equation (13), P_i_ and P_IL_, respectively, are the micropower i power and the microgrid load, and P_RS_ is the battery charging and discharging power; when P_RS_ > 0, the battery discharges; when P_RS_ < 0, the battery is charging.

Battery operation constraints

(14) $${SOC}_{\min}(t)\leq SOC(t)\leq {SOC}_{\max}(t)$$

In Equation (14), SOC(t) is the energy storage capacity at time t.

## 3. IMOHHO-GWO

Inspired by the HHO algorithm, the initial algorithm loop iteration simulates the cooperative hunting behavior of the Harris hawk group, including encirclement, raid, and pursuit strategies. Its core mechanism is to expand the range through random jumps and decentralized search in the exploration phase and to dynamically adjust the strategy according to the prey’s escape energy (E) in the development phase, with the advantages of flexible strategy switching, strong global search capability, and few parameters. However, it has the disadvantages of insufficient convergence speed for high-dimensional and complex problems and is easily affected by parameter sensitivity in the development phase. The GWO algorithm is inspired by the pack hunting mechanism of the grey wolf social hierarchy. Its core mechanism guides the group to move toward the optimal region through α, β, and δ positions, and the position update formula relies on leadership information, emphasizing fast convergence. The advantages of the grey wolf algorithm include its simple algorithm structure, fast convergence speed, and easy implementation. However, the disadvantage is that it easily falls into local optima, and the global exploration ability is weak.

### 3.1. Convergence Strategy of HHO and GWO

a. Social hierarchy to guide the search direction: Using GWO’s α, β, and δ wolves to define the leadership and guide the population to move toward the potential optimal area.
b. Dynamic hunting strategy to enhance exploitation: Under the control of HHO’s escape energy (E), the GWO’s position update mechanism is introduced to optimize the local exploitation accuracy.
c. Hybrid exploration mechanism: Combining the random raid strategy of HHO with the group collaboration of GWO in the global search phase to enhance diversity.

**Figure 2:**
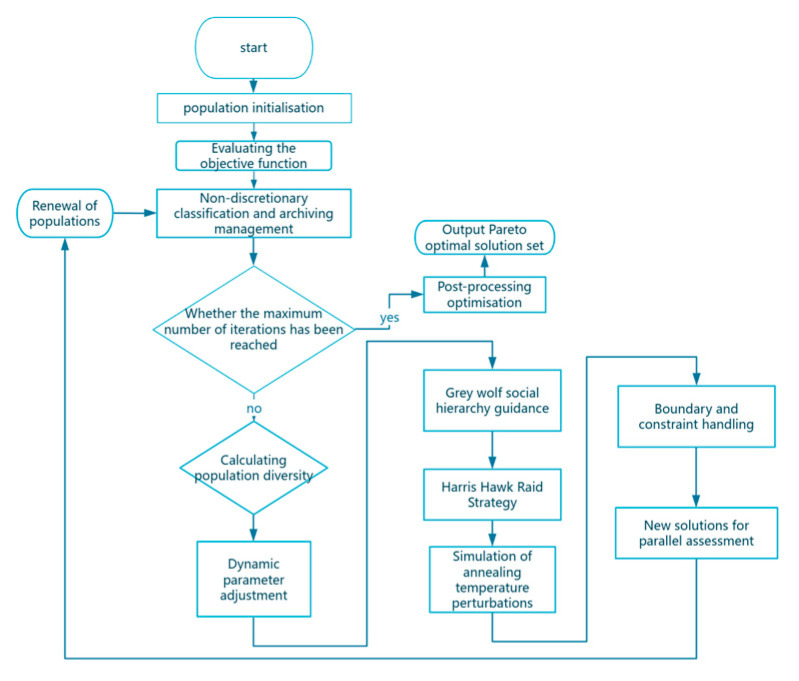
IMOHHOGWO algorithm flow chart (number of iterations: 500).

Its global search ability is significantly improved by the GWO algorithm’s leadership (α, β, and δ) to provide a clear search direction to avoid blind dispersion; the HHO algorithm’s random raid strategy expands the search range [32,33,34], especially for the multi-peak problem, to effectively avoid premature convergence. Local development efficiency and accuracy are optimized; the escape energy (E) in algorithm HHO is used to dynamically switch the development strategy (e.g., swoop trapping) to improve the local search adaptability; and the development direction is refined in the GWO algorithm’s leader position information to accelerate the convergence to a high-quality solution [35,36]. The fusion effect balances the development intensity and computational efficiency by precisely adjusting the strategy at the late convergence stage. The convergence speed and stability are enhanced, and the fast convergence characteristic of the GWO relies on α, β, and δ to rapidly approach the optimal region at the early stage. The dynamic perturbation mechanism of HHO jumps out of the local optimum through random perturbation at the late stage to avoid stagnation. The robustness and adaptability are enhanced, and the multi-strategy compatibility of the algorithm after the fusion is applicable to high-dimensional, multi-peak, nonlinear, and local search adaptations and multi-peak, nonlinear, and dynamic optimization problems. There is reduced parameter sensitivity and dependence on the HHO escape energy parameter through GWO’s social hierarchy; escape energy (E) and leadership synergy occur at high E, and stochastic exploration of HHO is dominated, combined with GWO’s group collaboration, to expand the search scope. At low E, GWO leadership guides exploitation, supplemented by HHO’s raid strategy, to refine the search. Figure 2 shows the flowchart of the IMOHHOGWO algorithm.

### 3.2. Improved Algorithm

#### 3.2.1. Nonlinear Factor

For the slow convergence of traditional linear particles, the traditional convergence factor (15) is improved to an exponential form (16).

(15) $$a=2-\frac{2t}{T}$$

(16) $$a=2e^{-\frac{3}{T}}$$

#### 3.2.2. Diversity Perception Energy Factor

The standard deviation of population diversity, σ, is introduced to dynamically adjust the energy factor to enhance local exploitation when the distribution of the solution set is dense. σ decreases when E is small, and global exploration is enhanced when it is sparse.

(17) $$E=2E_{0}(1-\frac{t}{T})(1-\frac{\sigma }{2\sigma_{\max}})$$

#### 3.2.3. Grey Wolf Social Hierarchy Guidance + Harris Hawk Raids

The Grey Wolf Optimization (GWO) algorithm is based on the social hierarchical structure and collaborative hunting behavior of wolf packs, and its core advantages are reflected in the following two aspects. The code is shown in Algorithm 1; the collaborative search mechanism, the leader guides (α, β, and δ), and the optimal three solutions (α wolf, β wolf, and δ wolf) jointly guide the population to move toward the potentially optimal region to form a collaborative search pattern, in which the position update formula is

(18) $$X(t+1)=\frac{X_{1}+X_{2}+X_{3}}{3}$$

**Algorithm 1.** GWO social hierarchy guidance.**Input:** Current individual position *X*, alpha, beta, delta positions 1: Calculate coefficient *a* = 2 × exp(−3 × *iter*/*maxIter*) 2: **For** each leader (alpha, beta, delta) **do** 3: | Generate random vectors *A* and *C* 4: | Calculate distance *D* = |*C* × leader.position—*X*| 5: | Update position component *X_leader* = leader.position—*A* × *D* 6: **end For** 7: **Output** *GWOPosition* = (*X_alpha* + *X_beta* + *X_delta*)/3

The HHO algorithm simulates the cooperative hunting behavior of Harris hawks and offers distinct advantages through its adaptive search mechanism. The algorithm’s core strength lies in its dynamic phase-switching capabilities, controlled by the energy factor (E), which governs the transition between the exploration and exploitation phases throughout the iterative process (see Algorithm 2).

During the exploration phase (|E| ≥ 1), the algorithm performs global stochastic search to maximize coverage of the solution space. In the exploitation phase (|E| < 1), HHO employs four distinct hunting strategies—soft siege, hard siege, progressive rapid dives, and rapid dives—each adapted to specific optimization scenarios to enhance local search precision (see Algorithm 3).

To mitigate premature convergence, the algorithm incorporates Lévy flight mechanics during the exploitation phase (see Algorithm 4). This mechanism enables random long-distance jumps, thereby improving the algorithm’s ability to escape local optima and explore promising regions of the search space.
**Algorithm 2.** Harris hawk exploration behavior.**Input:** current position *X*, alpha position, population mean 1: Generate random value *q* 2: **if** *q* ≥ 0.5 3: | Apply random escape pattern 4: | *newPosition* = alpha.position - rand × |alpha.position - 2 × rand × *X*| 5: **else** 6: | Apply perching strategy 7: | *newPosition* = (alpha.position - mean(population)) + rand × (varMax - varMin) 8: **Output** *newPosition*

**Algorithm 3.** Harris hawk exploitation with progressive rapid dives.**Input:** current position *X*, *GWOPosition*, energy *E* 1: Generate random value *r* 2: **if** *r* ≥ 0.5 and |*E*| < 0.5 3: | Apply soft besiege 4: | *newPosition* = *GWOPosition* - *E* × |*GWOPosition* - *X*| 5: **else** 6: | Apply hard besiege with progressive rapid dives 7: | *newPosition* = *GWOPosition* - *E* × |*GWOPosition* - *X*| + 0.01 × LevyFlight(*D*) 8: **Output** *newPosition*

**Algorithm 4.** Lévy flight.**Input:** dimension *nVar* 1: Set Lévy exponent *β* = 1.5//typical value for Lévy flight 2: Calculate sigma parameter: 3: | *numerator* = Γ(1 + *β*) × sin(π × *β*/2) 4: | *denominator* = Γ((1 + *β*)/2) × *β* × 2^((*β*-1)/2) 5: | *σ* = (*numerator*/*denominator*)^(1/*β*)//Lévy distribution parameter 6: Generate random variables: 7: | *u* = randn(1, *nVar*) × *σ*//numerator random variable 8: | *v* = randn(1, *nVar*)//denominator random variable 9: Calculate Levy flight step: 10: | **For** *i* = 1 to *nVar* **do** 11: | | *step*[*i*] = *u*[*i*]/|*v*[*i*]|^(1/*β*)//Lévy flight formula 12: | **end for** 13: **Output** *step*

#### 3.2.4. Simulated Annealing Perturbations

A temperature decay perturbation is applied with a probability of 0.3 during the development phase, and the code is shown in Algorithm 5.

(19) $$x_{\mathrm{new}}=x+T\cdot \Delta x,\;T=1-\frac{t}{T}$$

**Algorithm 5.** Simulated annealing perturbation mechanism.**Input:** current position *position*, current iteration *iter*, maximum iterations *maxIter*, variable bounds *varMin* and *varMax*, dimension *nVar* 1: Calculate temperature factor *T* = 1 - *iter*/*maxIter*//linear cooling schedule 2: Generate Gaussian noise *noise* = randn(*nVar*)//standard normal distribution 3: Calculate perturbation magnitude *magnitude* = *T* × (*varMax* - *varMin*) 4: Apply perturbation *perturbedPosition* = *position* + *magnitude* × *noise* 5: **For** *i* = 1 **to** *nVar* **do**//boundary handling 6: | **If** *perturbedPosition*[*i*] < *varMin* **then** 7: | | *perturbedPosition*[*i*] = *varMin* 8: | **If**
*perturbedPosition*[*i*] > *varMax* **then** 9: | | *perturbedPosition*[*i*] = *varMax* 10: end for 11: **Output** *perturbedPosition*

#### 3.2.5. ZDT1-3 Test Functions

The ZDT (Zitzler–Deb–Thiele) benchmark suite is a set of multi-objective optimization problems used to evaluate the performance of evolutionary algorithms. ZDT1, ZDT2, and ZDT3 each present different levels of complexity in their Pareto fronts, with ZDT1 offering a smooth and convex front, ZDT2 introducing local minima, and ZDT3 featuring a highly complex and non-convex front. To assess algorithm performance, two key indicators are used: IGD (Inverted Generational Distance), which measures the distance between generated solutions and the true Pareto front (with lower values indicating better convergence), and GD (Generational Distance), which calculates the average distance from generated solutions to the closest point on the true Pareto front (with smaller values indicating better convergence and diversity). These indicators help evaluate the ability of algorithms to balance convergence and diversity in multi-objective optimization tasks. Table 1 shows the ZDT1-3 test function.

IMOHHOGWO achieves efficient synergy between global exploration and local exploitation of multi-objective optimization problems by combining the social hierarchy steering mechanism of the Grey Wolf Optimizer (GWO) with the dynamic raid strategy of Harris Hawk Optimization (HHO). The grey wolf’s social hierarchy (α, β, and δ wolves synergistically guided) ensures that the algorithm converges to the potentially optimal region quickly, while the Harris hawks’ energy factor control strategy (dynamically switching between the exploration and exploitation phases) and the Lévy flight perturbation enhance the diversity of the solution and the quality of local optimal escapes. The fused algorithm further introduces adaptive parameter tuning (nonlinear convergence factor and diversity-aware energy factor) and a hybrid local search strategy (simulated annealing perturbation), which significantly improves the complex constraint handling capability and solution set distributivity.

**Figure 3:**
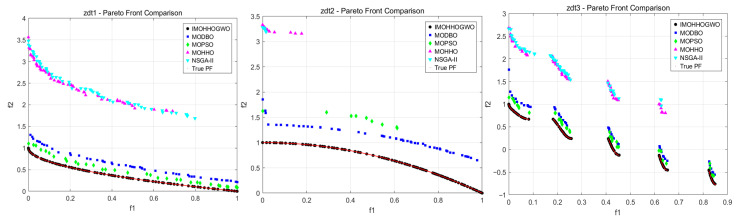
ZDT1-3 Pareto front comparison.

As shown in Figure 3 and Table 2, the experimental results demonstrate that the proposed IMOHHOGWO algorithm exhibits superior performance across all evaluation metrics on the ZDT test suite. Specifically, IMOHHOGWO achieved the lowest IGD values. The algorithm consistently obtained the best GD values, with particularly notable performance on ZDT2, indicating excellent convergence to the true Pareto front. Furthermore, IMOHHOGWO achieved the highest HV values among all algorithms, while MOHHO and NSGA-II failed to generate valid hypervolume measurements. The spacing metric results indicate competitive distribution uniformity, outperforming both MOHHO and NSGA-II. These comprehensive results, supported by the Pareto front visualizations showing superior coverage and convergence characteristics, validate that IMOHHOGWO significantly outperforms state-of-the-art multi-objective optimization algorithms in terms of convergence, diversity, and solution quality, establishing it as a highly effective approach for solving complex multi-objective optimization problems.

## 4. Background

This paper uses MATLAB2021a programming. The computer processor is as follows: the AMD Ryzen 9 7945HX processor features 16 cores, with each core supporting 2 threads, resulting in a total of 32 threads. It has a base clock speed of 2.5 GHz and can boost up to 5.4 GHz. The processor also includes 16 MB of L2 cache and 64 MB of L3 cache. As for RAM, the system supports DDR5 memory, with speeds of up to 5200 MHz, and can be configured with a maximum of 32 GB (2 × 16 GB) of RAM. In order to verify the above model, this paper analyzes a real microgrid wind system, at the same time, in order to improve the utilization of renewable energy, the operating costs of each distributed power source in the microgrid are shown in Table 3, and the greenhouse gas emission and treatment parameters of the micropower source are shown in Table 4. Battery parameters are shown in Table 5. The typical day 24 h factory data are shown in Table 6. The parameters of each distributed power source in the microgrid are shown in Table 7.

## 5. Results and Discussion

### 5.1. Simulation Analysis

**Figure 4:**
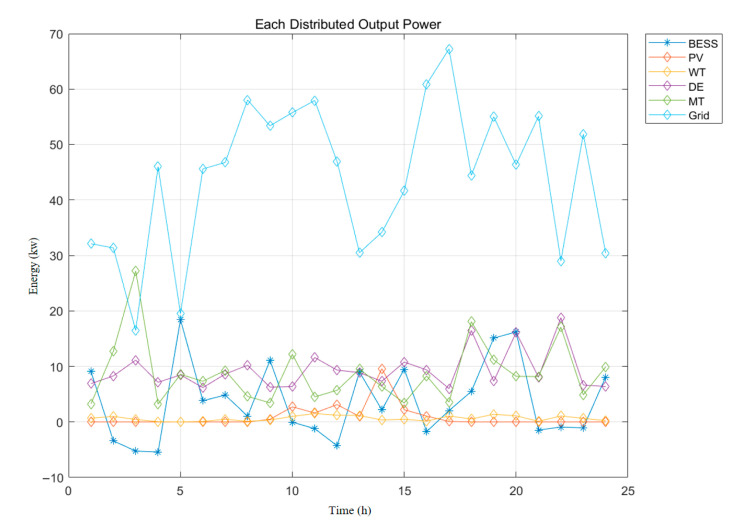
Each distributed output power.

**Figure 5:**
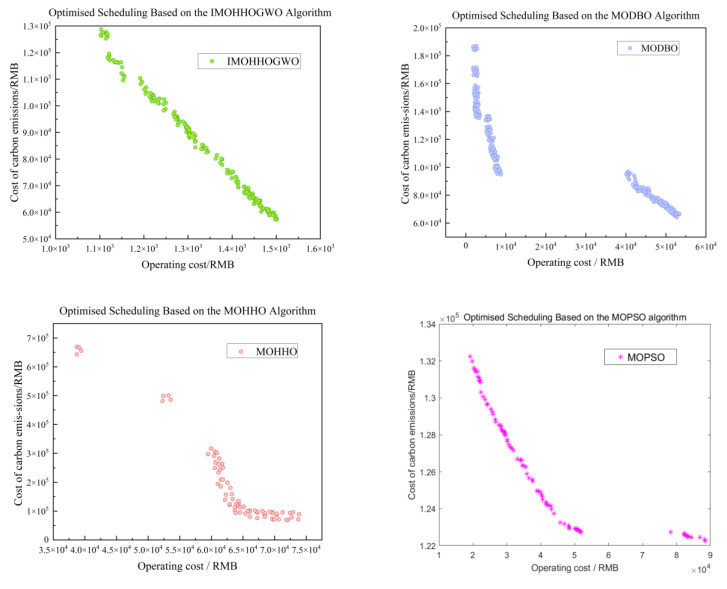
Iteration Curves of Four Algorithms.

In order to achieve the optimal scheduling of microgrid economy and environmental protection, this paper uses the IMOHHOGWO algorithm to iterate for 500 generations to generate 100 Pareto optimal solutions and obtains the optimal frontier of operation costs and environmental costs. From this, it can be seen that, with the increase in the operation costs of the microgrid, the pollutant treatment costs of the microgrid are relatively low. The relationship between carbon emission costs and operation costs is contradictory, and only a compromise between the two can be chosen. In order to highlight the advantages of the hybrid fusion algorithm, MOHHO, MODBO, and MOPSO are added to the experiments to compare the experiments; IMOHHOGWO can be evenly distributed on the Pareto frontier, and some of the particles are closer to the optimal solution compared with the other three algorithms. Our algorithm is significantly stronger than the other three algorithms in terms of convergence accuracy, convergence speed, local search, and robustness; each distributed output power is shown in Figure 4, the algorithm convergence curve is shown in Figure 5, its multi-objective scheduling is shown in Table 8.

In Table 9, according to the test results, the IMOHHOGWO iteration duration approximates 75 s. In microgrid optimization scheduling, the primary criteria for evaluating algorithms are their problem-solving time and memory requirements. Compared to the other three algorithms, the IMOHHOGWO algorithm demonstrates outstanding performance in computational speed. It significantly reduces operational costs in daily power scheduling while concurrently minimizing carbon emissions. Given the current global warming context, this algorithmic model offers substantial engineering benefits.

**Figure 6:**
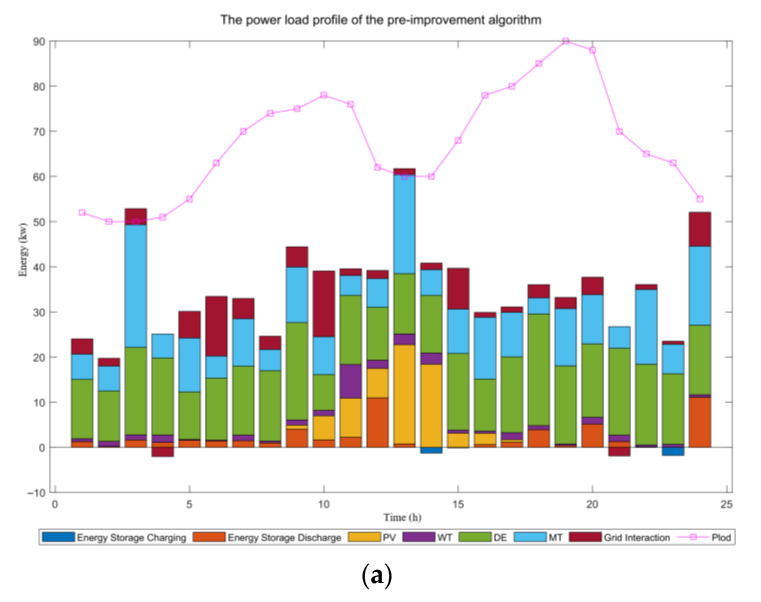

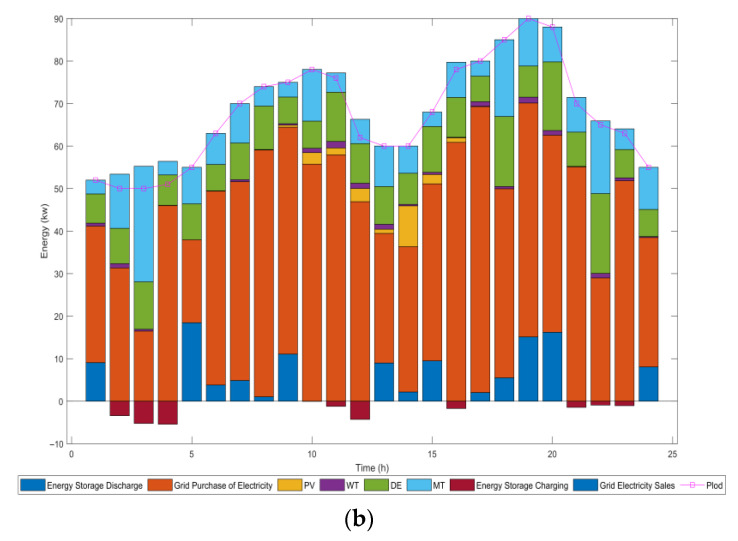
(**a**) Power load profile of the pre-improvement algorithm, and (**b**) power load profile of the hybrid intelligence algorithm.

Figure 6 clearly demonstrates the significant advantages of the improved hybrid intelligent algorithm for microgrid optimization scheduling by comparing the power load distribution diagrams of the pre-improvement algorithm (a) with the improved version (b). The enhanced hybrid intelligent algorithm exhibits more proactive, economical, and environmentally friendly scheduling strategies. Its key advantages are as follows: First, it significantly increases the consumption rate of renewable energy sources such as photovoltaic and wind power, almost entirely eliminating curtailment, thereby reducing reliance on fossil fuels from the outset. Second, the algorithm optimizes the scheduling of energy storage systems, making them more intelligent and efficient. It precisely charges during off-peak hours or when renewable energy generation exceeds demand, and discharges during peak hours, effectively achieving “peak shaving and valley filling”, thus greatly improving system stability and economic efficiency. Furthermore, the interaction strategy with the main grid becomes more economically rational, not only reducing expensive electricity purchases during peak periods but also creating additional revenue by selling excess electricity to the grid during periods of high renewable energy output. Lastly, the algorithm optimizes the output of traditional units, such as diesel generators and microturbines, ensuring that they serve only as efficient supplements or backup power sources. This significantly lowers operational costs and carbon emissions. In summary, this hybrid intelligent algorithm successfully achieves the dual optimization of economic efficiency and environmental sustainability in microgrid systems, demonstrating its immense value in improving overall system performance.

### 5.2. Single-Objective Optimization

A total of two different scenarios were chosen to compare the impact of single-objective function optimizations on the optimal scheduling of microgrids when running the model: finding the solution when operating costs are the lowest, and finding the solution when carbon emissions are the lowest.

**Figure 7:**
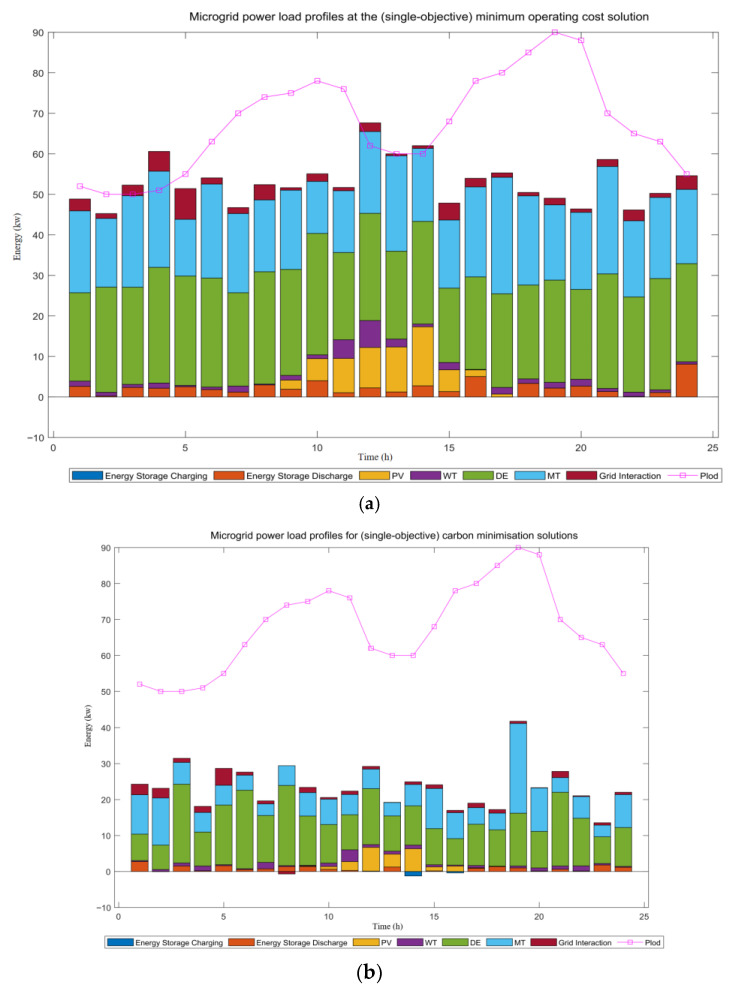
(**a**) Microgrid power load profiles at the (single-objective) minimum operating cost solution, and (**b**) microgrid power load profiles for (single-objective) carbon minimization solutions.

As shown in Figure 7, fluctuations in the operating conditions of the power system at different ambient temperatures lead to significant variations in the load distribution scheme. When a single economic metric is used as the optimization objective, the system will prioritize dispatching diesel units for baseload supply. This is due to the high generation cost of gas-fired units compared to diesel units. However, in a multi-objective optimization model that incorporates carbon emissions, the share of diesel units in operation will be strictly limited due to their high carbon emission factor. In a single economic target model, energy storage primarily functions for power regulation. Its charging and discharging strategy is closely linked to the tariff mechanism: charging during off-peak periods and discharging during peak tariff intervals. This bidirectional adjustment mechanism effectively reduces the system’s overall costs. However, it is important to note that the current output level of photovoltaic (PV) power systems still falls short of fully meeting the overall power demand.

Under the multi-objective optimization framework, both carbon emission costs and operating costs impose dual constraints on the system’s dispatch decisions. The combined effect of economic indicators and environmental governance costs plays a crucial role in shaping the microgrid’s energy dispatch strategy. When carbon emissions are the sole optimization objective, system operation exhibits the following characteristics: the frequency of diesel units and energy storage usage is strictly limited, while the output of gas-fired units and utility power purchases increases. This shift results from the economic disparities in the environmental cost system, where the environmental management cost of gas-fired units is significantly lower than that of diesel units. Notably, gas turbines have a clear advantage over diesel units and conventional thermal power in terms of sulfur dioxide (SO_2_) and nitrogen oxide (NO_2_) emissions. Since the cost of end-of-pipe treatment for pollutants like SO_2_ and NO_2_ is two orders of magnitude higher than CO_2_ emission costs, gas-fired power generation proves to be more environmentally friendly.

### 5.3. Multi-Objective Optimization Scheduling vs. Single-Objective Optimization Scheduling

As shown in Table 10, the comparison of different optimization approaches for microgrid scheduling reveals distinct advantages. The multi-objective optimization approach balances both costs and carbon emissions, optimizing the use of renewable energy while ensuring grid stability and cost savings. It efficiently employs energy storage for peak shaving and reduces reliance on fossil fuels, making it the most effective solution for both economic and environmental goals. In contrast, single-objective economic optimization focuses primarily on cost reduction, often relying on fossil fuel-based generation during low renewable output, which leads to higher emissions and less efficient use of renewable resources. On the other hand, single-objective carbon emission minimization maximizes the use of renewables to reduce emissions, but it may result in higher costs due to less flexible dispatch of backup generation. Overall, the multi-objective optimization approach offers the most balanced and efficient solution, integrating both economic benefits and environmental sustainability.

## 6. Conclusions

In this study, the optimal scheduling problem of microgrid operation is analyzed in detail, and based on the dual demands of the economy and environmental protection in microgrid scheduling, a dual-objective optimization model of comprehensive operating costs and carbon emissions is established. In order to solve the shortcomings of the traditional grey wolf algorithm, which is prone to premature convergence, the Harris Hawk Optimization algorithm is innovatively integrated with the grey wolf algorithm to form a hybrid intelligent optimization method. The proposed hybrid algorithm strengthens its global exploration capabilities by introducing a dynamic adaptive mechanism and adopts the Pareto frontier optimization strategy to obtain a non-inferior solution set. Simulation experiments verify that the method improves the convergence speed by 18% compared with the MOHHO algorithm and achieves a 97.1% reduction in operating costs, a 91.4% reduction in carbon emission costs, and a 22% improvement in the uniformity of the distribution of the Pareto solution set in the microgrid test case. This study proves that the hybrid strategy has significant advantages in multi-objective optimization, especially in solving the problem of conflicting objectives; shows good adaptability; and is applicable to engineering practice.

## Figures and Tables

**Table 1:** ZDT1-3 test function.

Test Function	f1	f2
ZDT1	$minf_{1}\left(x_{1}\right)=x_{1}$	$minf_{2}(x)=g\left(1-\sqrt{\left(f_{1}/g\right)}\right)$ $g(x)=1+9\sum_{i=2}^{m}x_{i}/\left(m-1\right)$ $s.t.\;0\leq x_{i}\leq 1,\;i=1, 2,\ldots ,\;30,\;m=30$
ZDT2	$minf_{1}\left(x_{1}\right)=x_{1}$	$minf_{2}(x)=g\left(1-{\left(f_{1}/g\right)}^{2}\right)$ $g(x)=1+9\sum_{i=2}^{m}x_{i}/\left(m-1\right)$ $s.t.\;0\leq x_{i}\leq 1,\;i=1, 2,\ldots ,\;30,m=30$
ZDT3	$minf_{1}\left(x_{1}\right)=x_{1}$	$minf_{2}(x)=g\left(1-\sqrt{f_{1}/g}-\left(f_{1}/g\right)sin\left(10\pi f_{1}\right)\right)$ $g(x)=1+9\sum_{i=2}^{m}x_{i}/\left(m-1\right)$ $s.t.\;0\leq x_{i}\leq 1,\;i=1, 2,\ldots 30,\;m=30$

**Table 2:** Evaluation indicators.

ZDT Test Function	Algorithm	IGD	GD	HV	Spacing
ZDT1	IMOHHOGWO	0.005457483	0.000160994	0.718245734	0.010915841
	MODBO	0.105478098	0.0134272	0.586794498	0.01778148
	MOPSO	0.015991113	0.001027113	0.701984641	0.019467523
	MOHHO	0.971229064	0.149448205	0	0.072009007
	NSGA-II	1.030093286	0.136262293	0	0.023899979
ZDT2	IMOHHOGWO	0.005366116	4.77685E−05	0.443433903	0.010412705
	MODBO	0.351724216	0.050378461	0.109103142	0.033920912
	MOPSO	0.708313239	0.190107182	0	0.099084718
	MOHHO	2.526116922	0.59851304	0	0.016399538
	NSGA-II	2.582003529	0.917594038	0	0.00900713
ZDT3	IMOHHOGWO	0.006075159	0.000242328	0.601626492	0.00919141
	MODBO	0.126822825	0.022813843	0.523296438	0.068080756
	MOPSO	0.084408876	0.011523584	0.562794384	0.030188536
	MOHHO	0.772131651	0.115864593	0.090405316	0.017544413
	NSGA-II	0.872717681	0.137291279	0.040347006	0.0244188

**Table 3:** Operating costs.

Typology	Management Costs (RMB/KW h)	Service Life/Year
PV	0.0096	20
WT	0.0296	10
MT	0.0401	10
DE	0.0859	10

**Table 4:** Pollutant emission factors and costs.

Type of Pollutant	Pollutant Emission Factor (g/KW h)		Cost of Governance (RMB/KG)
	DE	MT	
${CO}_{2}$	1.400	1.600	0.092
${SO}_{2}$	21.800	0.440	27.540
${NO}_{2}$	0.454	0.008	6.490

**Table 5:** Energy storage parameters.

Typology	Parametric	Numerical Value	Parametric	Numerical Value
BESS	Maximum capacity/KW h	150	Initial capacity/KW h	50
	Minimum capacity/KW h	5	Maximum output power/KW	50
	Maximum Input Power/KW	30	Charge and Discharge Rate	0.9

**Table 6:** **The** 24 h factory data.

Electrical Loads (MW)	PV (MW)	WT (MW)	Purchase Price of Electricity from the Grid (KW h)	Price of Electricity Sold to the Grid (KW h)
52	0	2	0.38	0.36
50	0	2	0.38	0.36
50	0	2	0.38	0.36
51	0	2	0.38	0.36
55	0	2	0.38	0.36
63	0	1	0.38	0.36
70	0	2	0.82	0.36
74	0	1	0.82	0.36
75	4	2	0.82	0.36
78	6	3	1.35	0.36
76	10	8	1.35	0.36
62	12	10	1.35	0.36
60	23	4	1.35	0.36
60	20	3	1.35	0.36
68	6	2	0.82	0.36
78	4	1	0.82	0.36
80	1	2	0.82	0.36
85	0	2	1.35	0.36
90	0	2	1.35	0.36
88	0	3	1.35	0.36
70	0	2.5	1.35	0.36
65	0	2.5	1.35	0.36
63	0	2	0.38	0.36
55	0	1	0.38	0.36

**Table 7:** Equipment parameters.

Equipment Name	Parameters
DE	6–30 KW
MT	3–30 KW
Grid	−60–60 KW

**Table 8:** Optimized scheduling using the IMOHHOGWO algorithm.

Algorithm	Dispatch Type	Operating Costs/RMB	Cost of Carbon Emissions/RMB
IMOHHOGWO	Economic Dispatch	1114.1	124,809.2
	Environmental Scheduling	1500.6	57,027.2
	Multi-target scheduling	[1114.1, 1500.6]	[57,027.2, 124,809.2]
MODBO	Economic dispatch	2570.1	155,121.9
	Environmental scheduling	5406.2	96,022.5
	Multi-target scheduling	[2570.1, 5406.2]	[69,411.8, 155,121.9]
MOHHO	Economic dispatch	38,726.4	669,346.4
	Environmental scheduling	73,867.9	69,411.8
	Multi-target scheduling	[38,726.4, 73,867.9]	[96,022.5, 669,346.4]
MOPSO	Economic dispatch	119,135	132,227
	Environmental scheduling	88,187.5	122,340
	Multi-target scheduling	[119,135, 88,187.5]	[122,340, 132,227]

**Table 9:** IMOHHOGWO vs. other algorithms.

Algorithm	Time Reduction	Improved Convergence Speed	Increased Pareto Density	Reduced Operating Cost	Reduced Carbon Emission Costs
MODBO	12%	15%	20%	56.7%	19.5%
MOHHO	15%	18%	22%	97.1%	91.4%
MOPSO	20%	25%	15%	99.1%	14.5%

**Table 10:** Microgrids optimization scheduling (multi-objective vs. single-objective).

Feature	Multi-Objective Optimization	Single-Objective (Economic)	Single-Objective (Carbon Emissions)
Energy Storage Utilization	Balanced (charging and discharging)	Primarily discharge, with limited charging	Balanced but higher emphasis on discharge
Renewable Energy Usage (PV/WT)	Maximized when available	Limited by economic factors	Maximized to reduce emissions
Grid Interaction	Optimized (minimized during peak)	Increased during peak	Increased during low renewable output
Backup Generation (DE/MT)	Minimally used	Relatively high during low renewables	Used minimally to reduce emissions
Cost Efficiency	Balanced with environmental goals	High, but not environmentally optimized	Higher due to reliance on renewables
Carbon Emissions	Reduced due to efficient renewable use	Higher, especially during low renewables	Minimizing emissions is the priority

## Data Availability

All data generated or analyzed during this study are included in this published article.
